# Identification of 4 autophagy-related genes in heart failure by bioinformatics analysis and machine learning

**DOI:** 10.3389/fcvm.2024.1247079

**Published:** 2024-01-29

**Authors:** Xiwei Deng, Ziqi Yang, Tongzheng Li, Yang Wang, Qinchuan Yang, Rui An, Jian Xu

**Affiliations:** ^1^ Department of Radiology, Xijing Hospital, Fourth Military Medical University, Xi’an, Shaanxi, China; ^2^ Department of Interventional Surgery Center, Xijing Hospital, Fourth Military Medical University, Xi’an, Shaanxi, China; ^3^ Department of Oncology, Bethune International Peace Hospital, Shijiazhuang, Hebei, China; ^4^ Department of Cardiology, Xijing Hospital, Fourth Military Medical University, Xi’an, Shaanxi, China; ^5^ Department of Gastrointestinal Surgery, Xijing Hospital, Fourth Military Medical University, Xi’an, Shaanxi, China

**Keywords:** heart failure, autophagy, key genes, bioinformatics analysis, machine learning

## Abstract

**Introduction:**

Autophagy refers to the process of breaking down and recycling damaged or unnecessary components within a cell to maintain cellular homeostasis. Heart failure (HF) is a severe medical condition that poses a serious threat to the patient's life. Autophagy is known to play a pivotal role in the pathogenesis of HF. However, our understanding of the specific mechanisms involved remains incomplete. Here, we identify autophagy-related genes (ARGs) associated with HF, which we believe will contribute to further comprehending the pathogenesis of HF.

**Methods:**

By searching the GEO (Gene Expression Omnibus) database, we found the GSE57338 dataset, which was related to HF. ARGs were obtained from the HADb and HAMdb databases. Annotation of GO and enrichment analysis of KEGG pathway were carried out on the differentially expressed ARGs (AR-DEGs). We employed machine learning algorithms to conduct a thorough screening of significant genes and validated these genes by analyzing external dataset GSE76701 and conducting mouse models experimentation. At last, immune infiltration analysis was conducted, target drugs were screened and a TF regulatory network was constructed.

**Results:**

Through processing the dataset with R language, we obtained a total of 442 DEGs. Additionally, we retrieved 803 ARGs from the database. The intersection of these two sets resulted in 15 AR-DEGs. Upon performing functional enrichment analysis, it was discovered that these genes exhibited significant enrichment in domains related to “regulation of cell growth”, “icosatetraenoic acid binding”, and “IL-17 signaling pathway”. After screening and verification, we ultimately identified 4 key genes. Finally, an analysis of immune infiltration illustrated significant discrepancies in 16 distinct types of immune cells between the HF and control group and up to 194 potential drugs and 16 TFs were identified based on the key genes.

**Discussion:**

In this study, TPCN1, MAP2K1, S100A9, and CD38 were considered as key autophagy-related genes in HF. With these relevant data, further exploration of the molecular mechanisms of autophagy in HF can be carried out.

## 1 Introduction

Heart failure (HF) is a multifaceted clinical syndrome that arises from a multitude of factors resulting in anomalous alterations to the structure and function of the heart. HF can be caused by a variety of factors, including myocardial infarction (MI), ischemic heart disease, cardiomyopathy, heart valve disease, hypertension, and arrhythmias. Myocardial ischemia is a prevalent contributor to HF and is closely associated with its high mortality rate (1). According to the 2021 American Heart Association Statistical Update, around 6 million individuals in the United States experience HF, representing approximately 1.8% of the entire populace. The incidence of HF is much higher among the elderly, with a prevalence rate of 4.3% in the 65–70 age group in 2012. It is expected to steadily increase and reach a prevalence rate of 8.5% by 2030. In addition, the five-year mortality rate for HF is as high as 50%, indicating a poor prognosis (2). The heightened incidence, hospitalization, and mortality rates of HF necessitate a more thorough exploration and understanding of its pathogenesis. In recent years, increasing evidences suggest that autophagy appears to be intricately linked with the development of cardiovascular disease and shows promise as a viable therapeutic target (3).

Autophagy is a biological degradation process within cells, which breaks down cellular components and reuses them. It also serves as a mechanism for cellular self-protection, enabling the elimination of pathogens, damaged proteins, organelles, and other items within cells, thereby maintaining normal cell function (4). Many diseases are linked to autophagy, including neurodegenerative diseases, cardiovascular disease, musculoskeletal disorders, lung diseases, kidney disease, metabolic syndrome, liver disease, cancer, and so on (5). As research on autophagy in cardiovascular diseases advances, it has become increasingly evident that this process plays a crucial role not only in maintaining heart morphology and function, but also in the development of HF. For example, moderate autophagy can delay the progression of HF. With ATG5 and ATG7 knockout in animal models, insufficient autophagy leads to increased hypertrophic cardiomyocytes, which promotes the deterioration of heart function (6). Overactive autophagy can accelerate the deterioration of heart function. In the final stage of HF, damaged organelles, ROS and other harmful factors accumulate in cardiomyocytes and lead to overactive autophagy, which damages important organelles and proteins while clearing harmful factors, thus accelerating the deterioration of heart function (7). At this point, downregulation of autophagy levels can play a protective role in the heart. Furthermore, autophagosome clearance is the final stage of the autophagy process, and inhibition of this function can lead to the accumulation of autophagosome, which has an adverse impact on the body (8). However, many autophagy-related genes for HF remain unknown and require further exploration.

**Figure 1:**
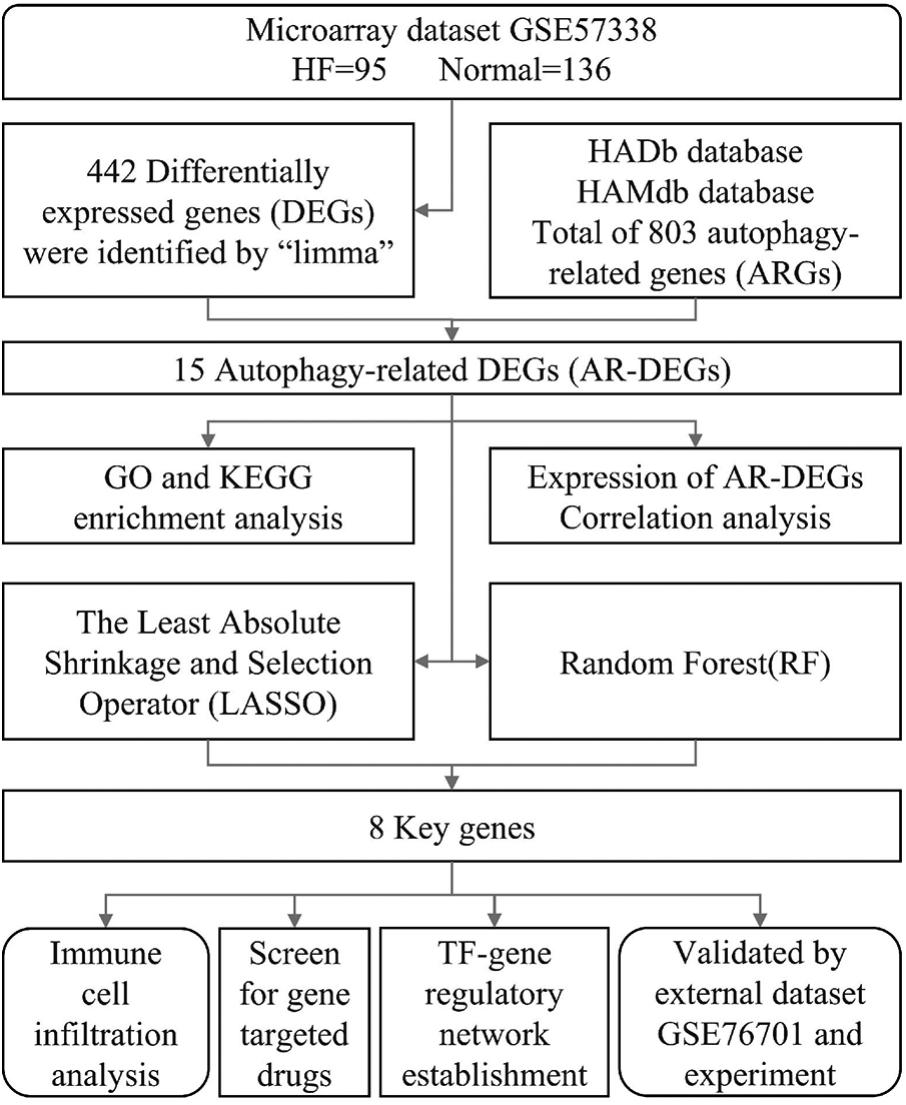
Flowchart of the current study.

In this study, a bioinformatics analysis method based on transcriptome sequencing was used to discover autophagy-related genes in HF patients, with the aim of providing fresh perspectives on the diagnosis and treatment methods. Figure 1 illustrates the workflow for the specific analysis.

## 2 Materials and methods

### 2.1 Data source

We downloaded the microarray dataset GSE57338 (mRNA) and GSE76701 (mRNA) related to heart failure on the Gene Expression Omnibus (GEO) database. GSE57338 came from GPL11532 platform [(HuGene-1_1-st) Affymetrix Human Gene 1.1 ST Array] and GSE76701 came from GPL570 platform [(HG-U133_Plus_2) Affymetrix Human Genome U133 Plus 2.0 Array]. The samples in GSE57338 and GSE76701 were both from human heart tissue. The GSE57338 dataset included 95 patients with HF and 136 normal individuals as controls; the GSE76701 dataset had 4 HF samples and 4 normal samples. After removing duplicate genes from two autophagy-related gene databases HADb (http://www.autophagy.lu/index.html) and HAMdb (http://hamdb.scbdd.com/) (9), 803 autophagy-related genes (ARGs) were acquired in total (Supplementary File S1).

### 2.2 Identification of DEGs

To identify differentially expressed genes (DEGs), we performed differential analysis between the HF and normal samples in GSE57338 dataset using the R package “limma” (version 3.54.2) (10). *P* < 0.05 and |log2 (fold change, FC)| > 0.5 were set as filtration criteria. R package “EnhancedVolcano” (version 1.16.0) and “pheatmap” (version 1.0.12) were utilized to visualize the expression of DEGs.

### 2.3 Identification of AR-DEGs

In order to obtain autophagy-related DEGs (AR-DEGs), we intersected 803 ARGs with the DEGs detected from the GSE57338 dataset. We created Venn diagrams using the Sangerbox tool (http://www.sangerbox.com/) (11) to visualize the overlap of genes. To demonstrate the expression of AR-DEGs in GSE57338, we used box plots generated by the R package “ggpubr” (version 0.6.0). We also performed correlation analysis of AR-DEGs and visualized the results using the R package “corrplot” (version 0.92).

### 2.4 GO and KEGG enrichment analysis

Gene Ontology (GO) enrichment analysis is a common technique that helps in identifying biological processes (BP), molecular functions (MF), and cellular components (CC) that are over-represented in a set of genes of interest. The KEGG (Kyoto Encyclopedia of Genes and Genomes) pathway analysis can help researchers to gain insights into the biological functions of a set of genes and to identify the key pathways or processes that are involved in a particular biological phenomenon. The R package “clusterProfiler” (version 4.6.2) (12) was used for GO and KEGG analyses of AR-DEGs.

### 2.5 Identification of key genes via machine learning

To further screen key genes, two machine learning algorithms—LASSO (the least absolute shrinkage and selection operator) and RF (random forest)—were adopted. Lasso regression is a type of regression analysis that performs variable selection and estimates the coefficients of a linear regression model, while also imposing a penalty on the size of the coefficients to avoid overfitting. The LASSO regression method was applied using the “glmnet” package (version 4.1.6) (13) in R, with a parameter α set to 1 to refine the model's qualities. This enabled the identification of AR-DEGs and the removal of irrelevant genes from consideration. RF is a decision-making algorithm that constructs multiple decision trees and combines their outputs for improved accuracy in classification and regression tasks (14). The R package “randomForest” (version 4.7.1.1) was used to perform RF analysis. Lastly, we identified the key genes by taking the intersection of the genes obtained through LASSO and RF.

### 2.6 Validation of key genes by analysing external dataset

We downloaded the GSE76701 dataset to validate the reliability of the key genes obtained previously. Wilcoxon rank-sum tests were performed to determine whether these genes exhibited differential expression between the HF and control groups. To visualize the results, we used the R package “ggpubr” to generate box plots.

### 2.7 Establishment of animal models

In this study we used male C57BL/6 WT mice aged 6–8 weeks and weighing 20 ± 2 g for experiments which were approved by the Animal Ethics Committee of the Air Force Medical University. The mice were housed in a temperature-controlled chamber (25 ± 2°C) for a 12-hour light/dark cycle, and provided free access to food and water. Subsequently, the mice were randomly allocated to either a HF group or a control group. The mice in control group were raised under normal diet while the HF group were anesthetized with isoflurane and implanted with a micro-pump filled with angiotensin II (Ang II) subcutaneously, which was continuously infused at a rate of 2 ug/Kg/min for 4 weeks. RT-qPCR was performed after model establishment in the 4th week. The present study quantified the expression levels of Anp, Bnp, and β-Mhc in two distinct sets of samples, with the simultaneous elevation of all three biomarkers indicating the presence of HF. In addition, echocardiography was performed in the 4th week. Left ventricular posterior wall dimension (LVPWd), left ventricular end diastolic dimension (LVEDD), and left ventricular end-systolic dimension (LVESD) were measured to calculate left ventricular ejection fraction (EF) and fractional shortening (FS). EF ≤ 50% is considered indicative of HF.

### 2.8 Validation of key genes by RT-qPCR

Expression of identified key genes were further validated by RT-qPCR which was performed using cDNA from the 4-week time point (HF = 3, normal = 3, C57BL/6 mice left ventricle). RNA was extracted using the TRIzol® Reagent and reverse transcription was conducted following the manufacturer's protocols (Yeasen Biotechnology, Shanghai, China). To quantify mRNA expression, the comparative quantification method (2−ΔΔCT) was employed, with normalization to the housekeeping gene Gapdh, utilizing the QuantStudio™ 5 system (Applied Biosystems, United States). Primer sequences used in this study can be found in Supplementary File S2.

### 2.9 Immune infiltration analysis

Gene Set Variation Analysis (GSVA) is a computational approach that leverages tissue gene expression profiles to calculate the scores of distinct immune cells, facilitating the analysis of disparities in immune gene sets between HF and normal samples (15). We also performed correlation analysis between the key genes and immune cells using the R package “corrplot”.

### 2.10 Target drug screening

The comparative toxicogenomics database (CTD) helped us to predict the potential gene target-based drug. CTD could be accessed by visiting NetworkAnalyst 3.0 platform (https://www.networkanalyst.ca/NetworkAnalyst/) (16).

### 2.11 Construction of TF-gene regulatory network

Transcription factors (TF) are proteins that play a crucial role in regulating gene expression by binding to specific DNA sequences and controlling the transcription process. The JASPAR database was used to identify TFs that bind to AR-DEGs in HF and it could also be accessed by visiting NetworkAnalyst 3.0 platform which can be used to generate an analysis of the TF-gene regulatory network. Cytoscape software (version 3.9.1) was utilized for visualization.

## 3 Results

### 3.1 Identification of DEGs

**Figure 2:**
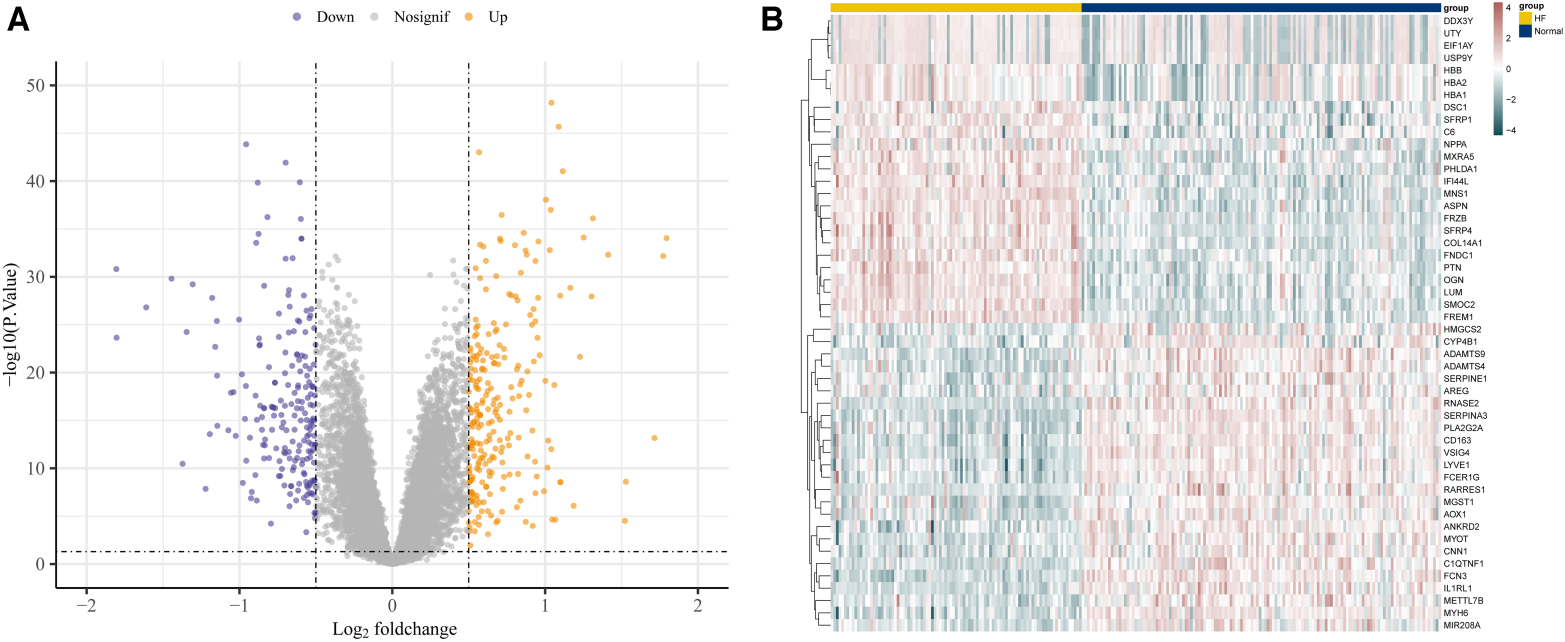
Identification of DEGs in GSE57338. (**A**) The volcano plots of DEGs. (**B**) Heat map results of the top 50 DEGs.

Utilizing the afore-mentioned filtration criteria [*P*-value <0.05 and | log2 (fold change, FC) | > 0.5], we identified 442 DEGs from the GSE57338 dataset, consisting of 241 up-regulated genes and 201 down-regulated genes (Supplementary File S3). The volcano plots of these DEGs are shown in Figure 2A and heat map results of the top 50 DEGs are shown in Figure 2B.

### 3.2 Identification of AR-DEGs

**Figure 3:**
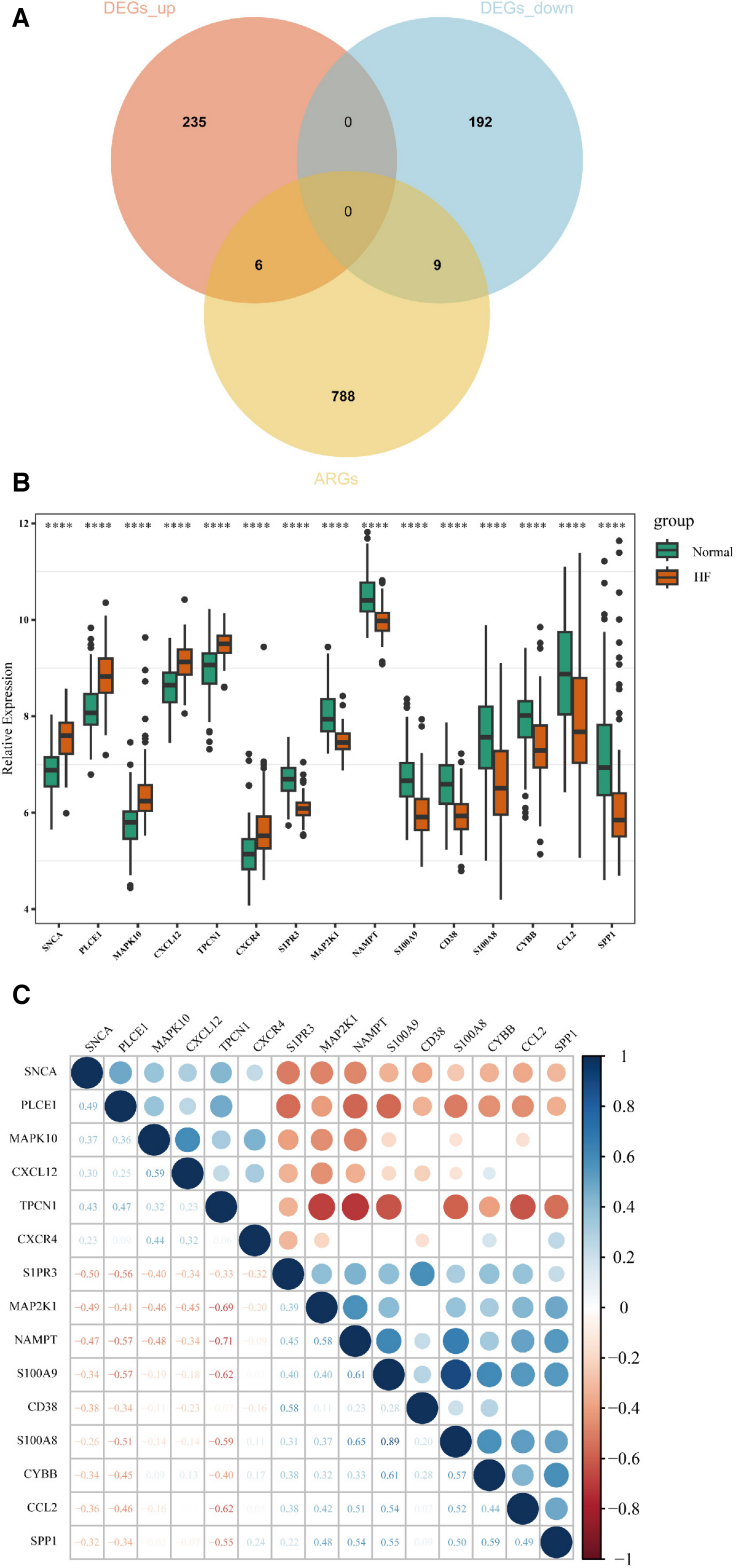
Identification of AR-DEGs in GSE57338. (**A**) Venn diagram of 241 up-regulated DEGs, 201 down-regulated DEGs and 803 ARGs. (**B**) Boxplot of 15 AR-DEGs in the HF and normal group. (**C**) Correlation heatmap of 15 AR-DEGs. The values of relative coefficients between genes with *P*-value <0.05 were marked; blue represented positive correlation and red represented negative correlation. The darker the color, the larger the absolute value of the correlation coefficient, indicating a stronger correlation.

A total of 15 AR-DEGs of HF were obtained by intersecting 241 up-regulated genes and 201 down-regulated genes with 803 ARGs, of which 6 up-regulated (SNCA, PLCE1, MAPK10, CXCL12, TPCN1, CXCR4) and 9 down-regulated (S1PR3, MAP2K1, NAMPT, S100A9, CD38, S100A8, CYBB, CCL2, SPP1), and the Venn diagram is shown in Figure 3A. The expression of 15 AR-DEGs in the HF group and the normal group were shown in Figure 3B. In the correlation matrix analysis, the values of relative coefficients between genes with *P*-value <0.05 were marked in Figure 3C.

### 3.3 Enrichment analysis of AR-DEGs

**Figure 4:**
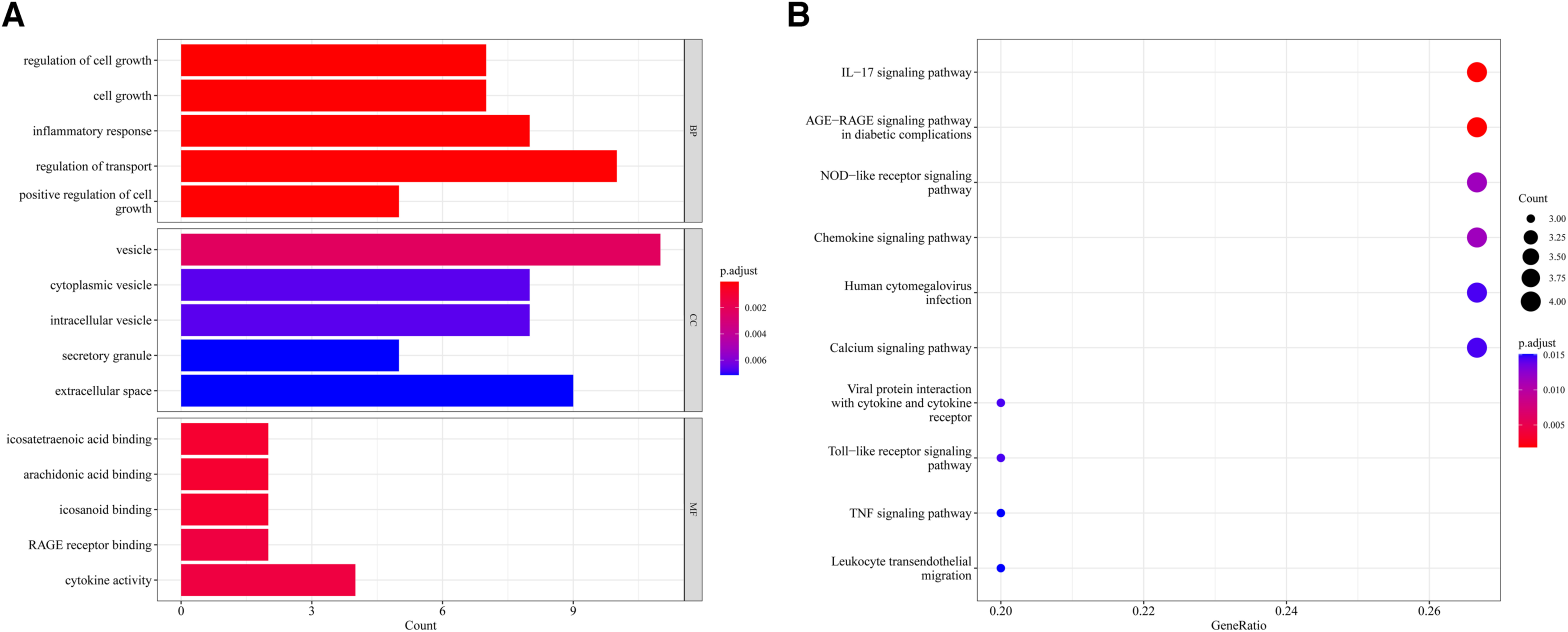
Enrichment analysis of 15 AR-DEGs. (**A**) Bar chart of Go enrichment results (Top 5). (**B**) Bubble plot of KEGG enrichment results (TOP10).

604 biological process (BP), 17 chromosomal location (CC), 37 molecular function (MF) of GO analysis and 148 KEGG signaling pathways were obtained by clustering AR-DEGs of HF with R package “clusterProfiler” (Supplementary Files S4,S5). Figures 4A,B show the top 5 enriched GO annotation terms and top10 KEGG pathways, respectively. For BP analysis, the top 3 significantly enriched terms are “regulation of cell growth”, “cell growth” and “inflammatory response”. In terms of the CC ontology, the AR-DEGs were predominantly localized within the following subcellular compartments: “vesicle,” “cytoplasmic vesicle,” and “intracellular vesicle”. MF analysis showed that “icosatetraenoic acid binding”, “arachidonic acid binding” and “icosanoid binding” were the most significant items. According to the KEGG analysis, the signal pathways were mainly enriched in the “IL-17 signaling pathway”, “AGE-RAGE signaling pathway in diabetic complications” and “NOD-like receptor signaling pathway”.

### 3.4 Machine learning screened for key genes of AR-DEGs in HF

**Figure 5:**
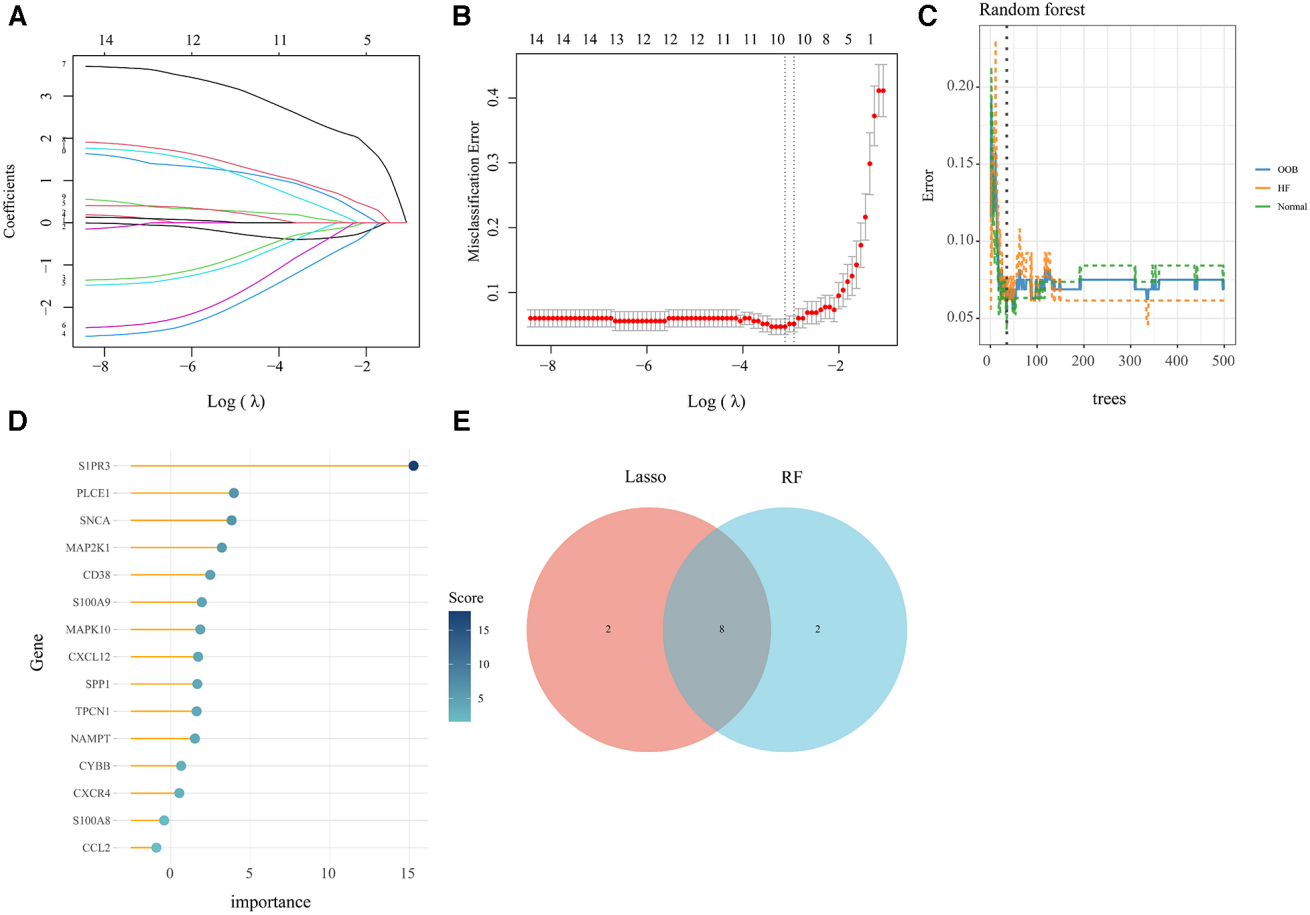
Machine learning screened for key genes of AR-DEs in HF. (**A,B**) Screening for key genes by LASSO regression model. The number of genes (*n* = 10) corresponding to the lowest point of the curve is the most suitable. (**C,D**) The RF algorithm showed the error in HF and normal group and genes are ranked based on the importance score. (**E**) Venn diagram showed 8 key genes were identified via the above two algorithms.

In order to obtain key genes in HF, we utilized the expression matrices of 15 AR-DEGs to construct the best diagnostic model via both LASSO regression and RF algorithms. The LASSO regression algorithm further narrowed down their range and obtained a total of 10 variables as key AR-DEGs (Figures 5A,B) (Supplementary File 6). The RF algorithm prioritized the 15 AR-DEGs by quantifying the importance of each gene (Figures 5C,D) (Supplementary File S7). We took the top 10 ranked genes based on their scores and intersected them with the 10 genes obtained from the Lasso algorithm. This resulted in a final set of 8 genes (SNCA, MAPK10, CXCL12, TPCN1, S1PR3, MAP2K1, S100A9, CD38) (Figure 5E).

### 3.5 Validated by external dataset and experiment

**Figure 6:**
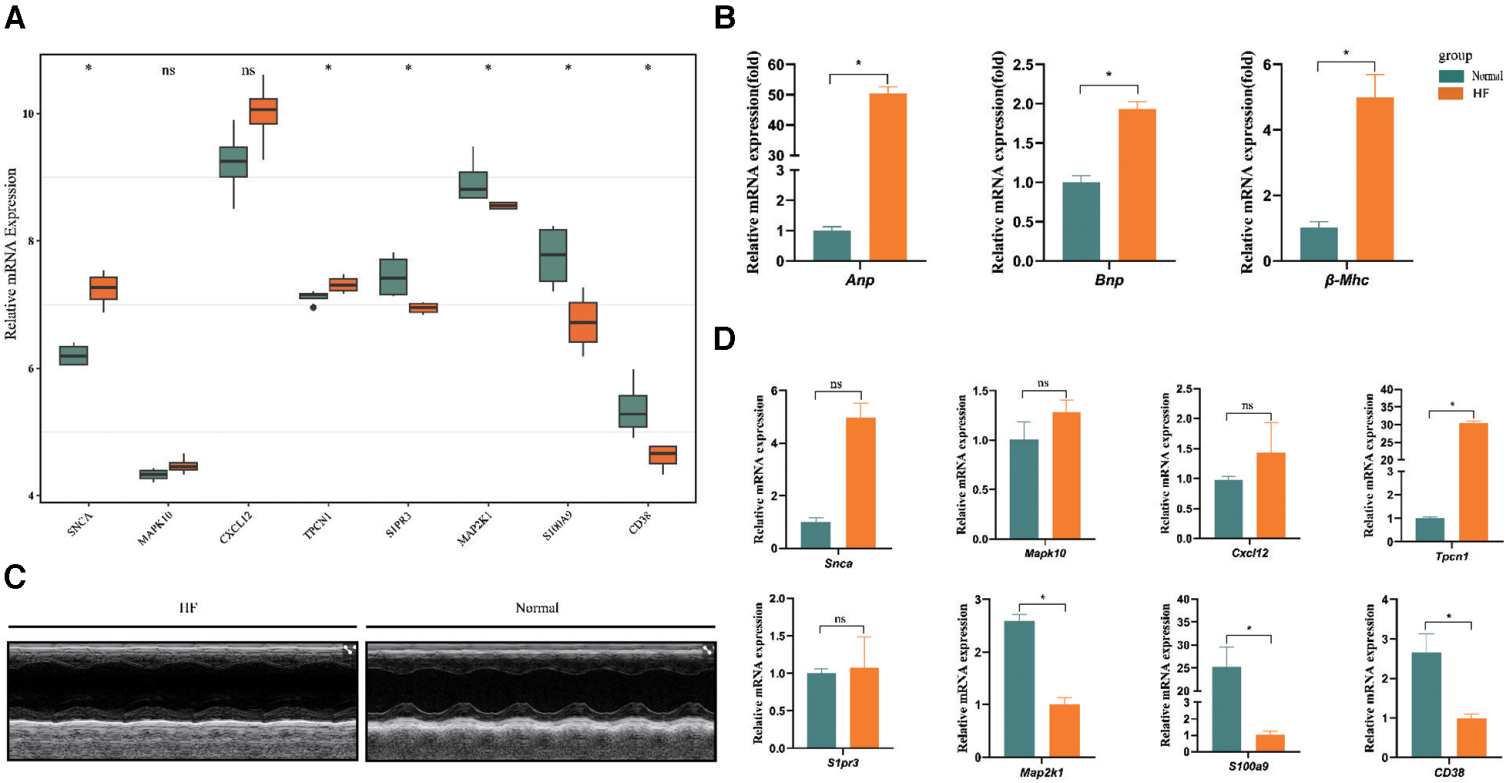
Further validation of expression of 8 key genes. (**A**) Validation by dataset GSE76701. (**B**) The expression levels of Anp, Bnp, and β-Mhc in HF and normal sample. (**C**) Representative echocardiographic images taken 28d post-Ang II. (**D**) Validation of expression of key genes between samples from HF and normal mice (*n* = 3 per group) by RT-qPCR. *P < 0.05.

In the dataset GSE76701, we utilized the Wilcoxon test methodology to identify high expression of SNCA and TPCN1 in the HF group, while S1PR3, MAP2K1, S100A9, and CD38 showed lower expression. These findings were completely consistent with the expression trend observed in GSE57338. However, we did not find a significant difference in the expression of MAPK10 and CXCL12 between HF and control groups (Figure 6A). In the animal experiment, the relative mRNA expression levels of Anp, Bnp, and β-Mhc were significantly higher in the HF group compared to the Normal group (Figure 6B). Following the calculation of echocardiographic data, the EF of mice in the HF group was only around 33%, whereas the normal group exhibited an EF of approximately 64%. These findings collectively confirmed the successful construction of the HF model (Figure 6C) (Supplementary File S8). We found high expression of Tpcn1 in the HF group, while Map2k1, S100a9, and Cd38 showed lower expression levels that were consistent with the expression trend observed previously in GSE57338. However, we observed no significant difference between HF and normal groups in the expression of Snca, Mapk10, Cxcl12, and S1pr3 (Figure 6D). The inconsistency in the expression of these genes across external datasets and animal experiments may be attributed to the potential inter-species differences in the pathogenesis of the corresponding genes. Therefore, further empirical investigation is imperative to shed more light on this matter. Subsequently, we intended to conduct further studies centered on the 4 differentially expressed key genes with significance: Tpcn1, Map2k1, S100a9 and Cd38.

### 3.6 Immunoinfiltration analysis

**Figure 7:**
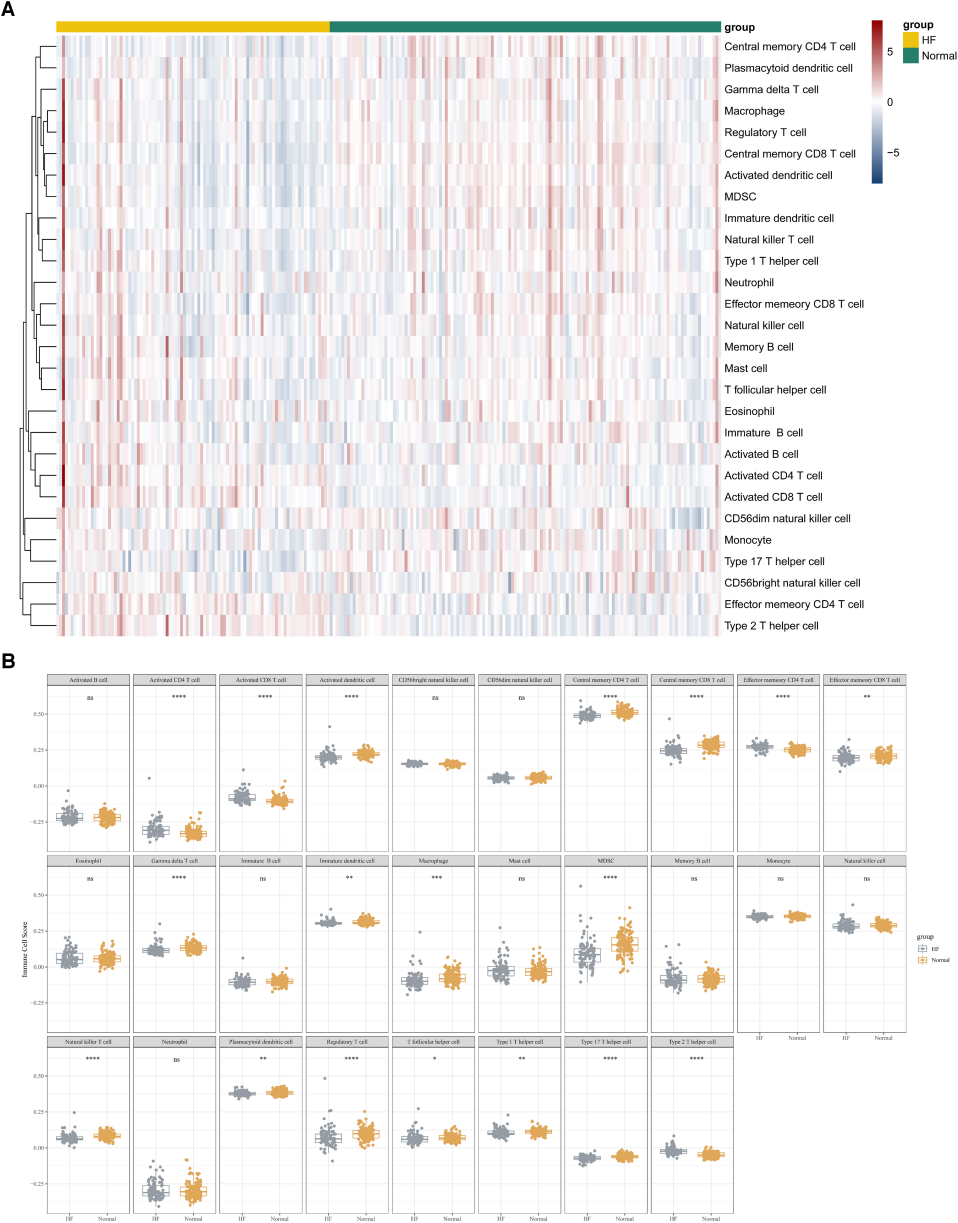
ssGSEA algorithm used to assess the level of immune infiltration between the HF and normal group in GSE57338. (**A**) Heat map results of the 28 kinds of immune cells in each sample. (**B**) Facet boxplot results of the 28 kinds of immune cells in the HF and normal group.

**Figure 8:**
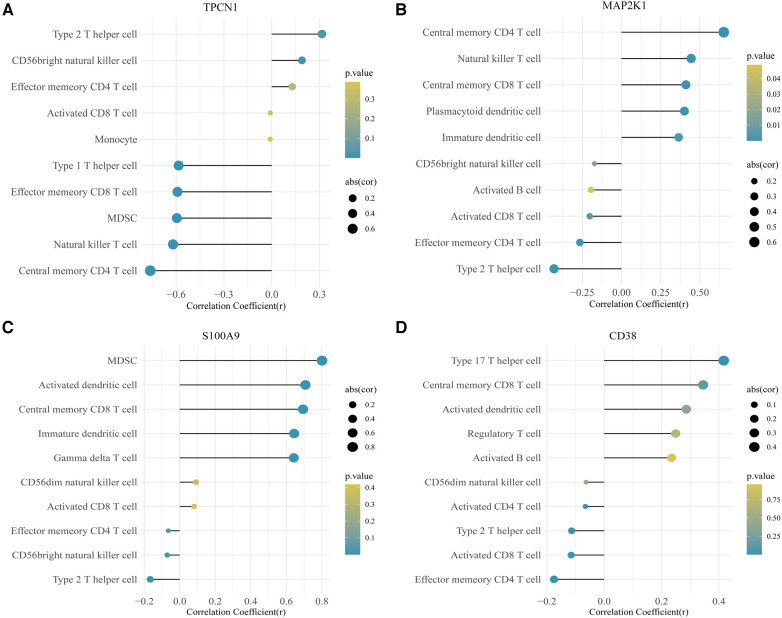
Correlation plot for 4 key genes and the top 10 immune gene sets. (**A**) TPCN1. (**B**) MAP2K1. (**C**) S100A9. (**D**) CD38.

In this study, we adopted the ssGSEA (single-sample gene set enrichment analysis) algorithm to evaluate the level of immunoinfiltration of these 28 immune cells in samples from HF and normal groups and the results were visualized in the heat map (Figure 7A).The faceted boxplot demonstrated that HF patients had a higher level of activated CD4 T cell, activated CD8 T cell, effector memory CD4 T cell and Type 2 T helper cell and a lower level of activated dendritic cell, central memory CD4 T cell, central memory CD8 T cell, effector memory CD8 T cell, gamma delta T cell, immature dendritic cell, macrophage, MDSC, natural killer T cell, plasmacytoid dendritic cell, regulatory T cell, T follicular helper cell, type 1 T helper cell, and type 17 T helper cell (Figure 7B). Figure 8 presents the correlation relationship between 4 key genes and top 10 differential immune gene sets in the form of a correlation plot. Here we only display the top five immune cells that show a positive correlation with each gene, as well as the top five immune cells that have a negative correlation with each gene. The complete data is available in Supplementary File S9.

### 3.7 Screening for gene targeted drugs

The CTD database was used to screen gene targeted drugs associated with the 4 key genes for HF in the NetworkAnalyst 3.0 platform, and 194 drugs predicted are shown in Supplementary File S10. Based on the degree of proportionality between gene-chemical associations, the analysis revealed that Cyclosporine and Estradiol exhibit strong binding to the HF gene.

### 3.8 Transcription factor (TF)-gene regulatory network establishment

**Figure 9:**
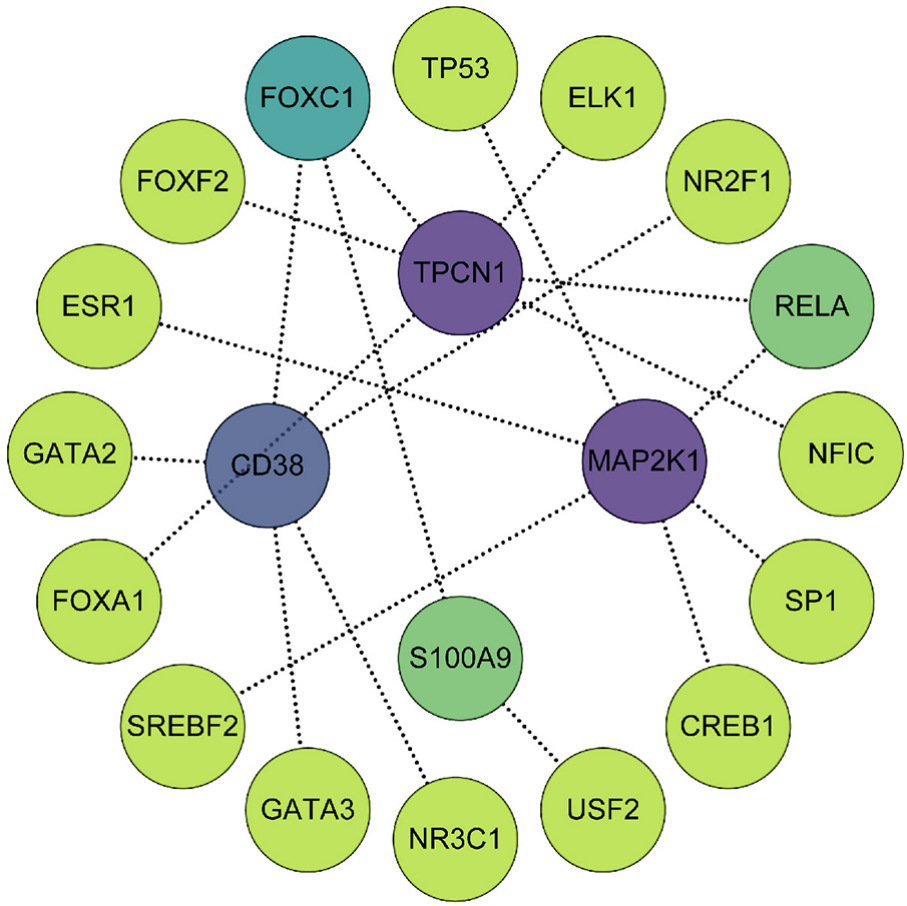
The TF-gene regulatory network consisted of 4 key genes, 16 transcription factors and 19 edges. The degree of correlation between TFs and genes were reflected by the depth of the color.

Using the JASPAR TF binding site database, we constructed a TF-gene regulatory network which included 20 nodes (4 seed genes and 16 transcription factors) and 19 edges based on 4 key genes in the NetworkAnalyst 3.0 platform. Among them, MAP2K1 and TPCN1 are each regulated by 6 TFs, whereas CD38 is regulated by 5 TFs and S100A9 by 2. The TF-gene regulatory network is visualized in Figure 9 and more detailed information can be obtained by referencing Supplementary File S11.

## 4 Discussion

HF constitutes the advanced phase of various cardiovascular disorders, such as cardiomyocyte injury and death, fibrosis and hypertrophy, inflammation reactions, neuroendocrine imbalance, etc. The management of HF predominantly involves symptomatic and neuroendocrine therapeutic approaches. Among them, neuroendocrine therapies, such as ACEI/ARB, β-blockers and aldosterone receptor antagonists, have been scientifically demonstrated to improve the prognosis of HF patients. However, the 5-year survival rate for HF patients remains suboptimal (17). Exploration of new intervention targets, pathways, and treatment methods for HF is necessary.

In the development of HF, autophagy dysfunction can lead to cardiomyocyte apoptosis, inflammatory response, and metabolic disorder, thus promoting the progression of HF. Currently, there is increasing research on the mechanism of HF and autophagy. Research has shown that in elderly mice, the insulin-like growth factor 1 receptor (IGF1R) can inhibit autophagic flux in the heart, leading to an increase in hypertrophic cardiomyocytes and hindering the recovery of heart function. However, low IGF1R activity can consistently improve aging heart function and myocardial bioenergetics in an autophagy-dependent manner. The IGF1R exhibits higher signal activity in HF of humans (18). In I/R injury, during ischemia, insufficient nutrient supply to myocardial cells can activate autophagy through AMPK, thereby maintaining energy production and promoting survival of myocardial cells during ischemia. However, prolonged ischemia can also suppress autophagic flux (19). During the reperfusion phase, autophagy flux can be restored, while the recovery of oxygen results in a noteworthy rise in the generation of ROS which also stimulates autophagy flux in cardiomyocytes and reduces cardiomyocytes loss and acute I/R injury (20). Every coin has two sides, the increase of ROS can also lead to an increase in BECLIN 1 expression, which will activate autophagic flux. This activation of autophagic flux is harmful and can lead to an increase in I/R injury (19). In pressure overload-induced HF, autophagy flux is also increased, and its role is both beneficial and harmful. When stressed, increased autophagy flux can exacerbate the production of myocardial fibrosis, leading to myocardial hypertrophy (21). On the contrary, there are studies proving that autophagy serves as an adaptive response to stress overload, during which AMPK or metformin can enhance autophagic response, thereby reducing myocardial hypertrophy (22). The above mechanisms elucidate that autophagy is a complicated process that exhibits notable dynamism. The regulation of autophagy, either up or down, is largely contingent upon the environmental factors that tissue cells encounter. The degree of adjustment can also lead to different outcomes, which poses many challenges for related research.

Our analysis commences at the level of gene, offering potential insights for subsequent research endeavors. Based on the GSE57338 dataset and 803 ARGs, we employed the R package “limma” to screen for differentially expressed genes and ultimately identified 4 key genes via a series of algorithms and subsequent verification processes.

TPCN1 (Two Pore Segment Channel 1) is a gene that encodes for a protein called two-pore channel 1. This protein belongs to the family of two-pore channels, which are ion channels found within the endolysosomal system of cells (23). TPCN1 is very important in regulating calcium ion homeostasis and lysosomal function, and has been linked to various physiological processes such as autophagy, apoptosis, and viral infection. The presence of TPC1 and TPC2 is critical to maintaining proper levels of basal and induced autophagy in cardiomyocytes. On the contrary, the lack of these proteins can lead to a decrease in cell viability under stressful conditions (24). Studies on the mechanism of TPCN1 in HF are not in-depth, but its effect on cardiomyocytes and its relationship with autophagy can furnish different directions on exploring HF.

The gene MAP2K1, responsible for encoding the MAP kinase kinase protein, belongs to the category of dual specificity protein kinases. This protein actively participates in the phosphorylation cascade of the mitogen-activated protein (MAP) kinase pathway. MAP kinases are integral to numerous cellular processes including cell proliferation, differentiation, survival, and apoptosis. MAP2K1 is also known as MEK1. In the signaling hierarchy of a cardiac myocyte, the MEK1-ERK1/2 pathway is likely to hold a central regulatory position (25). In a myocardial ischemia-reperfusion model, the activation of ERK1/2 has been found to reduce apoptosis caused by reperfusion injury, indicating that the MAP2K1 signaling pathway may provide cardioprotective effects (26). Furthermore, the Raf/MEK/ERK pathway is capable of regulating the expression levels of LC3B and SQSTM1/p62 within cells, which act as important markers for autophagy within cells (27).

S100A9 (S100 calcium binding protein A9) encodes for a protein called S100A9 or Calgranulin B which is expressed by various cells of the immune system and is involved in a range of biological processes, including chemotaxis, antimicrobial activity, and cell signaling. The translocation of S100A9 to the nucleus allows for the regulation of MDSC differentiation by IL-10 secreted by macrophages, thereby achieving the role of protecting against HF (28). In addition, S100A9 can directly induce autophagy and apoptosis (29, 30).

CD38 (CD38 molecule) encodes for a non-lineage-restricted, type II transmembrane glycoprotein and it also functions as an enzymatic ectoenzyme. CD38 knockout mice were observed to have a protective effect on the heart when subjected to ischemic/reperfusion injury. This protective mechanism operates through the activation of the antioxidative stress pathway mediated by SIRT1/FOXOs. CD38 also serves as a crucial factor in cardiac hypertrophy by inhibiting SIRT3 expression and activating the Ca^2+^-NFAT signaling pathway (31). Research has shown that overexpression of CD38 can downregulate the expression of Rab7 and its adaptor protein, pleckstrin homology domain-containing protein family member 1 (PLEKHM1). The loss of Rab7/PLEKHM1 impairs autophagosome-lysosome fusion, which leads to a blockade of autophagy flux and results in heart dysfunction under H/I conditions. These findings indicate that targeted inhibition of CD38 overexpression could be a promising therapeutic strategy (32).

Moreover, we performed enrichment analysis on 15 AR-DEGs. The results showed that significantly enriched GO terms were “regulation of cell growth”, “inflammatory response”, and “regulation of transport”, and signal pathways, such as “IL-17 signaling pathway”, “NOD-like receptor signaling pathway” and so on. Autophagy serves as a mechanism for clearing out dysfunctional or unnecessary materials within the cell and recycling them for energy production and the maintenance of cellular homeostasis, so it is essential in regulating cell growth and coping with various stresses such as starvation, infection, and inflammation (33). IL-17 (Interleukin-17) plays a vital role in regulating cardiac disorders. The concentration of IL-17 in the blood plasma was determined to be significantly elevated amongst individuals with HF in comparison to those without the condition. A negative correlation was found between the IL-17 levels and cardiac ejection fraction as well as fractional shortening. An increase in IL-17 disrupts calcium handling and cardiac remodeling via the NF-κB pathway, leading to impaired cardiac function. Inhibiting the IL-17 signaling pathway may become a potential treatment method for heart failure (34). In addition, IL-17 triggers autophagy by activating the ERK1/2-Beclin-1-p62 pathway, while suppresses through the BCL2-Beclin-1 and PI3K-GSK3β pathways. Conversely, autophagy suppresses IL-17 production by activating p38 MAPK signaling (35). The NOD-like receptor (NLR) signaling pathway is an essential factor within the innate immune system that detects and responds to microbial infection and cellular damage. NLRs are a group of endogenous cytosolic sensors responsible for detecting pathogen-related molecular patterns (PAMPs) and danger-associated molecular patterns (DAMPs). When activated, these receptors trigger a signaling cascade that ultimately leads to the secretion of pro-inflammatory cytokines, chemokines, and antimicrobial peptides (36). Many literatures indicated the NLR signaling pathway exerted a strong effect in the heart (37, 38). The vital role of the NLR pathway and its binding autophagy-related pathways in the pathological development of HF has been extensively studied (39, 40). These studies have offered insights that can be used to delve into the mechanistic aspects of autophagy-related HF.

Utilizing single-sample GSEA (ssGSEA), we investigated the extent of immune infiltration present in each sample according to a total of 28 immune cell types. Various T cells, including activated CD4 T cells, activated CD8 T cells, effector memory CD4 T cells, and Type 2 T helper cells, are positively correlated with the progression of HF, providing insight into the role of immune genes in the disease.

In addition, we constructed TF-gene regulatory networks and predicted target drugs, such as Cyclosporine and Estradiol based on 4 key genes of HF, which further expanded the scope of research and offered valuable insights for the development of novel drugs and precise clinical targeting therapies for HF.

This study is reliant on publicly accessible transcriptome information from the database, as well as the acquisition of autophagy-related genes from the same source. As the investigation into the autophagy phenomenon deepens, our understanding of the pathogenic mechanisms associated with autophagy increases, and new genes with autophagy regulatory functions are gradually being uncovered. However, due to the delay in updating the autophagy gene database, it is challenging to comprehensively include all autophagy-related genes in this study. Consequently, it is inevitable that an increasing number of positive genes will be overlooked over time, presenting a significant limitation that warrants further exploration in future research endeavors. In this study, animal modeling was employed, and subsequently, tissue specimens were extracted for gene testing. While the obtained results partially align with the validation findings from external datasets, it is undeniable that species variations can exert an influence on the ultimate outcomes. This constitutes another noteworthy limitation of this study. For future investigations, the collection of blood samples from HF patients for testing could be considered. This approach not only offers convenience in implementation and eliminates species differences but also holds potential for significant clinical translational implications. In addition, integrating the findings of this investigation with single-cell sequencing and advancements in multi-omics research is imperative. In future research endeavors, a more in-depth analysis should be conducted on the molecular mechanisms of the 4 pivotal genes. This analysis will be utilized to explore the diagnostic and prognostic potential of these genes, as well as enhance our comprehension of autophagy-related mechanisms involved in HF.

## 5 Conclusion

15 autophagy-related genes exhibiting differential expression in myocardial samples of patients with HF were ascertained using the GEO database. Furthermore, validation by the external dataset GSE76701 and mouse HF models underscored the importance of 4 key genes, namely Tpcn1, Map2k1, S100a9 and Cd38, in the pathogenesis and advancement of HF. The present findings suggest that the identified genes have potential utility as biomarkers or therapeutic targets for individuals with HF.

## Data Availability

Publicly available datasets were analyzed in this study. This data can be found here: https://www.ncbi.nlm.nih.gov/geo/query/acc.cgi?acc=GSE57338 https://www.ncbi.nlm.nih.gov/geo/query/acc.cgi?acc=GSE76701 Gene Expression Omnibus (GEO).
