# Supplementary material for: Identification of 4 autophagy-related genes in heart failure by bioinformatics analysis and machine learning
Source: Front Cardiovasc Med. 2024 Jan 29;11:1247079. doi: 10.3389/fcvm.2024.1247079 (PMC10859477; doi:10.3389/fcvm.2024.1247079)
Supplement: Supplementary file 12 [file Datasheet1.docx]

Supplementary Material

Identification of 4 autophagy-related genes in ischemic heart failure by bioinformatics analysis and machine learning

Xiwei Deng^1,2,3^, Ziqi Yang^1,2^, Tongzheng Li^4^, Yang Wang^3^, Qinchuan Yang^5^, Rui An^1,2*^, Jian Xu^1,2*^

*** Correspondence: Rui An,** [atczln@163.com](mailto:atczln@163.com); **Jian Xu,** xujian771939@163.com

## Supplementary Table

**Supplementary Table 1.** 803 autophagy-related genes.

**Supplementary Table 2.** The primer sequence information of qPCR experiment.

**Supplementary Table 3.**The detailed results of differential expression analysis.

**Supplementary Table 4.** The detailed results of GO analysis.

**Supplementary Table 5.** The detailed results of KEGG analysis.

**Supplementary Table 6.** 10 key AR-DEGs obtained by LASSO.

**Supplementary Table 7.** The importance score of 15 AR-DEGs by RF.

**Supplementary Table 8.** Representative parameters of mouse echocardiography.

**Supplementary Table 9.** Key genes and immune correlation dataset.

**Supplementary Table 10.** CTD database sources gene targeted drugs.

**Supplementary Table 11.** The details of TF‑gene regulatory network.
